# Association of relative brain age with tobacco smoking, alcohol consumption, and genetic variants

**DOI:** 10.1038/s41598-019-56089-4

**Published:** 2020-01-30

**Authors:** Kaida Ning, Lu Zhao, Will Matloff, Fengzhu Sun, Arthur W. Toga

**Affiliations:** 1 USC Stevens Neuroimaging and Informatics Institute, Keck School of Medicine of University of Southern California, Los Angeles, California 90033 USA; 2 Molecular and Computational Biology Program, University of Southern California, Los Angeles, CA 90089 USA; 3 Neuroscience Graduate Program, University of Southern California, Los Angeles, CA 90089 USA

**Keywords:** Biomarkers, Risk factors

## Abstract

Brain age is a metric that quantifies the degree of aging of a brain based on whole-brain anatomical characteristics. While associations between individual human brain regions and environmental or genetic factors have been investigated, how brain age is associated with those factors remains unclear. We investigated these associations using UK Biobank data. We first trained a statistical model for obtaining relative brain age (RBA), a metric describing a subject’s brain age relative to peers, based on whole-brain anatomical measurements, from training set subjects (n = 5,193). We then applied this model to evaluation set subjects (n = 12,115) and tested the association of RBA with tobacco smoking, alcohol consumption, and genetic variants. We found that daily or almost daily consumption of tobacco and alcohol were both significantly associated with increased RBA (P < 0.001). We also found SNPs significantly associated with RBA (p-value < 5E-8). The SNP most significantly associated with RBA is located in MAPT gene. Our results suggest that both environmental and genetic factors are associated with structural brain aging.

## Introduction

The number of American aged 65 and over is projected to reach 80 million by year 2050^1^. The brain aging process, while associated with structural changes, declined cognitive function, and increased risk of dementia, differs between individuals^2–4^. Therefore, to understand the factors associated with brain aging becomes increasingly important.

It is known that certain lifestyle habits are associated with accelerated atrophy in specific brain regions. Heavy smoking and heavy alcohol drinking are among the most studied adverse factors. Compared with non-smokers, smokers have significantly smaller grey matter volume and lower grey matter density in the frontal regions, the occipital lobe, and the temporal lobe. Further, smokers have a significantly greater rate of atrophy in regions that show morphological abnormalities in the early stages of Alzheimer’s disease^5–7^. It has also been reported that patients with alcohol use disorder show decreased regional grey and white matter volumes in the medial-prefrontal and orbitofrontal cortices. The loss of brain gray and white matter volume accelerates with aging in chronic alcoholics^8,9^. On the other hand, studies have shown that nicotine, a compound contained in tobacco, may improve attention and other cognitive functions in human subjects^10,11^. It has also been reported that drinking wine may be beneficial to the cardiovascular system, which is related to brain health^12,13^. To date, it is still unclear how smoking and alcohol consumption is associated with brain structural aging, especially when the morphology of all the brain regions is considered.

Besides lifestyle habits, genetic factors are also thought to be involved in brain aging. A recent study analyzed brain imaging data and chronological age (CA) information from twins and suggested that the brain aging process was heritable^14^. However, the extent to which individual genetic variants are associated with brain aging hasn’t been well studied, except for some conflicting results regarding the association between genetic variation in APOE, a gene associated with Alzheimer’s disease, and brain aging^15–17^. Therefore, we also investigated if genetic factors are associated with brain aging in addition to smoking and alcohol consumption. Further, genetic and environmental factors might interact in affecting traits, risk of complex diseases, or lifespan^18–20^. For example, hypertension interacts with APOE ɛ4 risk allele in affecting cognitive function^21^; the diabetes drug metformin extends lifespan of the rat model with hypertension, yet doesn’t extend lifespan of healthy rats^22^. Therefore, it is important to explore if there is genetic and environmental factor interaction in association with brain aging.

Recently, researchers have successfully used machine-learning methods to derive a biomarker that is commonly referred to as predicted brain age (PBA) or brain age based on brain imaging data. PBA reflects the degree of aging of the brain based on its anatomical characteristics, as computed based on brain morphology measurements across the entire brain. PBA has been derived and used in several studies, where the mean absolute error between PBA and CA was less than 5 years in adults^14,23,24^. Further, it has been shown that advanced brain age is associated with Alzheimer’s disease, objective cognitive impairment, and schizophrenia, etc.^23–27^. Before our research, many papers used the difference between PBA and CA (i.e., PBA - CA) for capturing deviation of person’s brain structural aging from norm^15,17^. However, due to regression dilution, this metric is correlated with CA and may not be optimal^28,29^. Therefore, we further developed a metric called relative brain age (RBA), which is independent of CA and indicates if a subject’s brain has experienced accelerated or decelerated aging compared to peers. While our manuscript was under review, Smith *et al*. independently reported a method for improving brain age delta estimation^29^. They gave statistical reasoning for the cause of the association between PBA - CA and CA in linear regression. They also suggested removing the association through stage 2 correction of brain age delta, which was very similar to our RBA metric. Further, since decline in cognitive function is associated with brain aging^30,31^, we investigated the correlation between RBA and cognitive function as a proof that RBA was able to capture brain aging deviation from norm.

In this study, we aim to quantify how smoking, alcohol consumption, and genetic variants are associated with RBA. We analyzed brain-imaging data collected for 17,308 UK Biobank subjects who were cognitively normal and were of European ancestry. We first trained a model that produces RBA using data for 30% of the subjects. We then applied the trained model to the remaining 70% of the subjects (i.e., the evaluation set) and obtained RBA for those subjects. We further studied the association of RBA with smoking, alcohol consumption, and genetic variants, as well as interaction among those factors, using the evaluation set subjects.

## Results

### Predicted brain age (PBA) and relative brain age (RBA)

**Figure 1:**
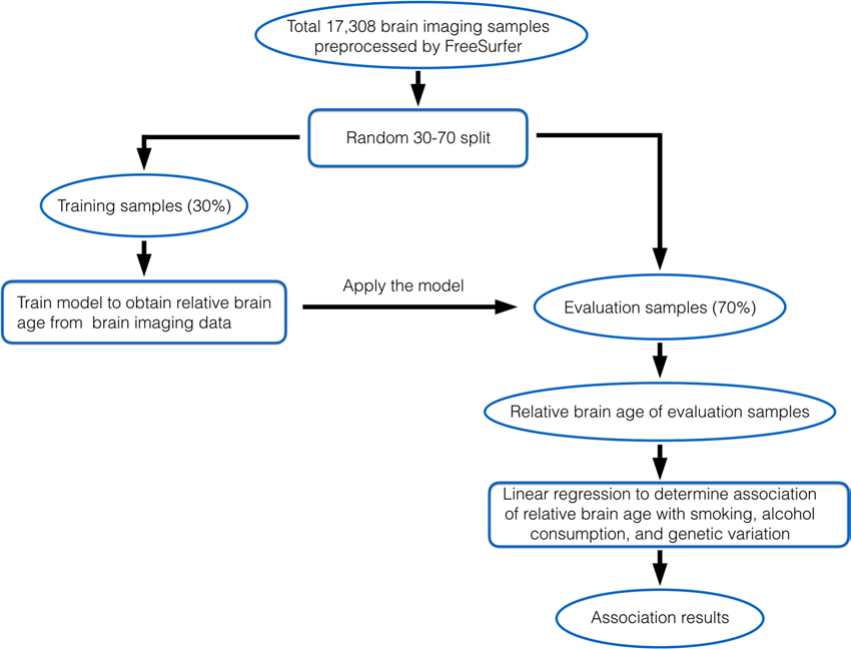
Procedure for studying the association of relative brain age with smoking, alcohol consumption, and genetic variation.

**Figure 2:**
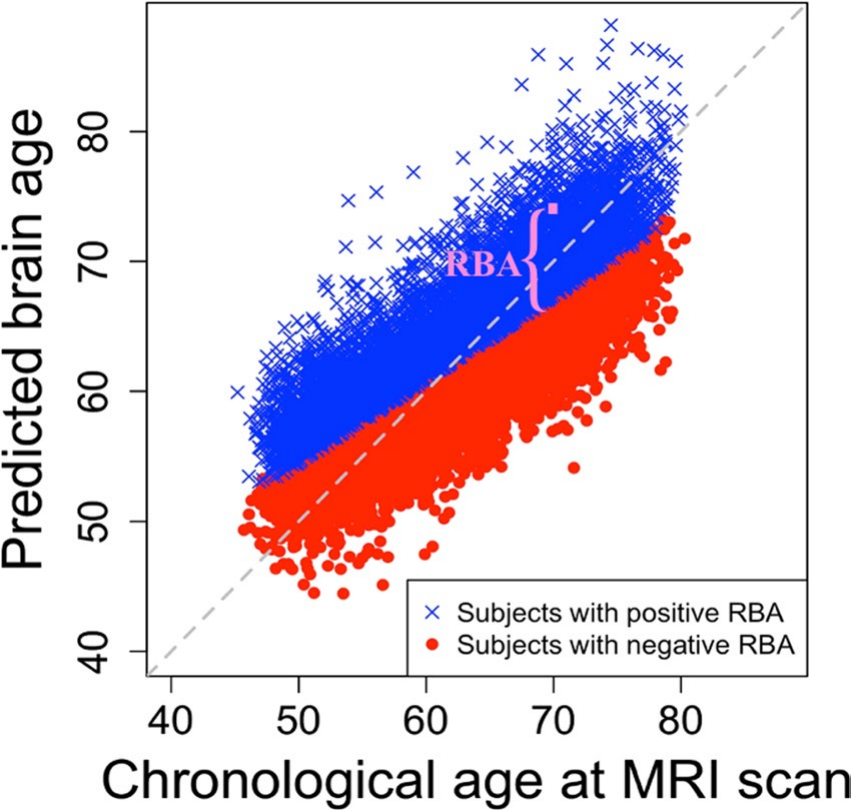
Relationship between chronological age and the predicted brain age. Subjects with positive relative brain age (RBA) are labeled with blue X’s; subjects with negative RBA are labeled with red dots. The RBA of a specific subject is illustrated in pink color.

We randomly split the data for 17,308 subjects with brain magnetic resonance imaging into training set (n = 5,193) and evaluation set (n = 12,115). Table 1 illustrates the demographic information for the subjects included in the training and evaluation sets. There was no significant difference in age, gender, smoking, and alcohol consumption (Supplementary Figs. 1, 2) between these two sets. We trained a model that produced the predicted brain age (PBA) and relative brain age (RBA) based on MRI measurements using training set subjects. We then applied this trained model to the evaluation set subjects (i.e., the evaluation set), and further obtained PBA and RBA for the evaluation set subjects (as illustrated in Fig. 1). The mean absolute error (MAE) between PBA and chronological age (CA) in the evaluation set was 3.8 years. The relationship between CA, PBA, and RBA for the evaluation set subjects is illustrated in Fig. 2 and Supplementary Figs. 3, 4. We carried out subsequent analyses using data of the evaluation set subjects.

**Table 1:** Demographic information for subjects included in the training and the evaluation data sets.

	Number of subjects	Male (%) | Female (%)	Age (mean [SD], min–max)
Training data (for model training)	5,193	2,466 (47%) | 2,727 (53%)	63.3 [7.4], 46.2–80.7
Evaluation data (for association analyses)	12,115	5,753 (47%) | 6,362 (53%)	63.3 [7.4], 45.2–80.3

### Cognitive function is negatively associated with RBA

**Figure 3:**
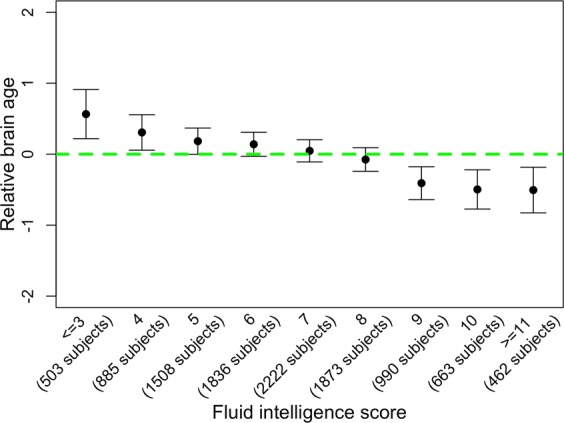
Relationship between Fluid intelligence score and relative brain age (RBA). Subjects with Fluid score of 0, 1, 2, and 3 (n = 28, 114, and 361, respectively) are grouped together. Subjects with Fluid score of 11, 12, and 13 (n = 334, 111, and 17, respectively) are grouped together, so that each group has more than 200 subjects.

Subjects who performed better in the cognitive tasks had a lower RBA than that of those who performed worse. As shown in Fig. 3, Fluid intelligence score was negatively associated with RBA (Spearman’s correlation = −0.07, p-value = 3E-13; R-squared = 0.005). Further, a lower RBA was associated with a better performance in memorizing a specific command and in memorizing the position of matching card pairs, and a lower response time in identifying matching cards. Detailed results on are shown in Supplementary Figs. 5–9.

### Previous tobacco smoking and alcohol consumption are significantly associated with RBA

**Figure 4:**
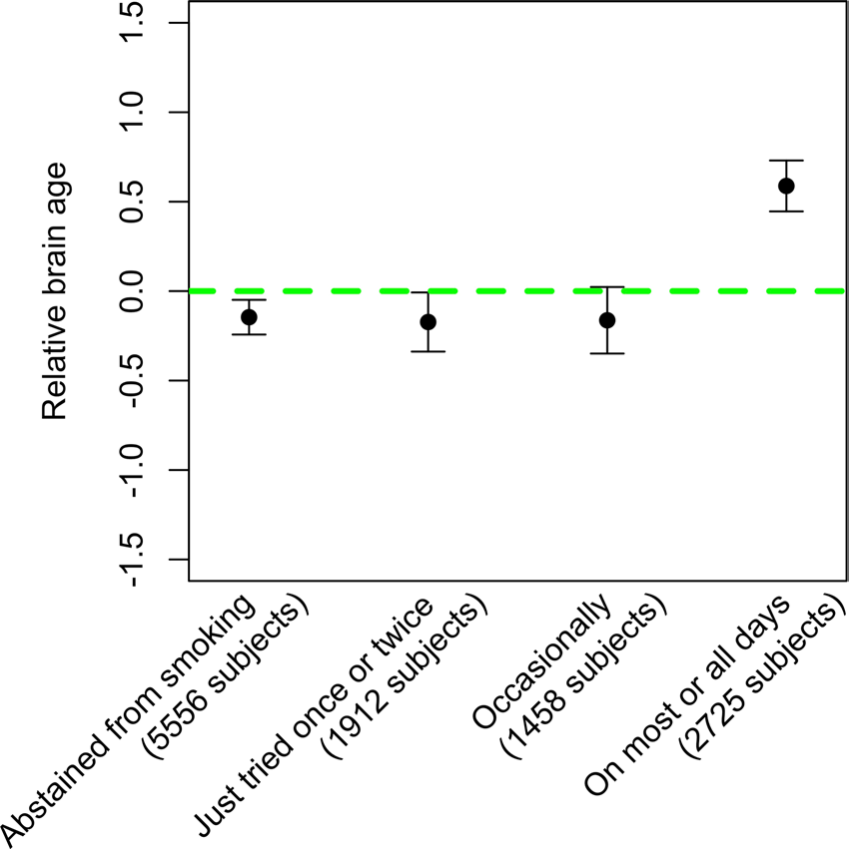
Relationship between previous tobacco smoking frequency and relative brain age.

Information of previous tobacco smoking frequency was collected for 11,651 of the evaluation set subjects during the visit for MRI scan. Regression analyses adjusting for gender and education showed that previous tobacco smoking frequency was statistically significantly associated with RBA (ANOVA F-test p-value < 2E-16, see Fig. 4). Pairwise comparisons showed that the most significant difference was between those who smoked on most or all days (with an average RBA of 0.6 years) and the rest of the smoking frequency categories (i.e., those who abstained from smoking, just tried once or twice, or occasionally), while there was no significant difference among the groups of subjects who didn’t smoke on most or all days (Supplementary Table 1).

**Figure 5:**
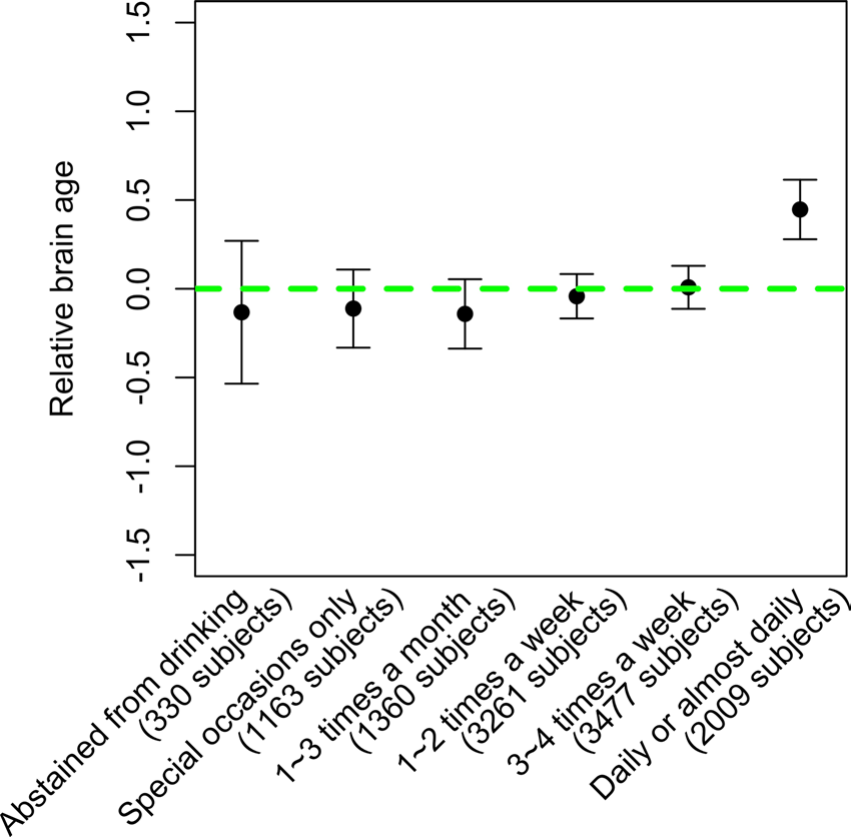
Relationship between alcohol intake frequency and relative brain age.

Information of current alcohol drinking frequency was collected for 11,600 of the evaluation set subjects during the visit for MRI scan. Regression analyses adjusting for gender and education showed that alcohol consumption frequency was statistically significantly associated with RBA (ANOVA F-test p-value = 9E-6, see Fig. 5). Pairwise comparisons among groups with different alcohol consumption frequencies showed that the strongest difference was between the group who drank alcohol on most or all days (with an RBA of 0.4 years) and the rest of the alcohol drinking frequency categories (i.e., those who abstained from drinking, drank at special occasions only, 1~3 times a month, 1~2 times a week, or 3~4 times a week), while the difference among groups who didn’t drink on most or all days was insignificant (Supplementary Table 2).

Smoking and alcohol consumption amount were positively correlated and had an additive effect on RBA. Among the 2,327 subjects who smoked on most or all days and did not abstain from alcohol, the correlation between the two variables was 0.08 (p-value = 9E-5). We used a regression model with RBA as the response variable and with smoking amount, alcohol consumption amount, sex, and education as predictors. According to this model, each additional pack-year of smoking was associated with 0.03 years of increased RBA (p-value = 2E-8); each additional gram of alcohol consumption per day was associated with 0.02 years of increased RBA (p-value = 6E-10). The R-squared value of this model was 0.032. As a comparison, a model with only smoking amount as predictor and adjusted for sex and education had an R-squared of 0.018. A model with only alcohol consumption amount as predictor and adjusted for sex and education had an R-squared of 0.015. We also built a regression model with an interaction term between alcohol drinking and smoking. The interaction term was insignificant, indicating that there was insufficient evidence to support the presence of an interaction between alcohol drinking and smoking in affecting RBA.

### Association between single nucleotide polymorphisms and RBA

**Figure 6:**
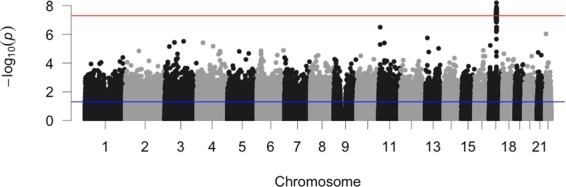
Manhattan plot for the association p-values between genetic variants and relative brain age across the genome. The red line indicates the genome-wide significant threshold on p-value (i.e., 5E-8). The blue line indicates p-value of 0.05.

We looked for single nucleotide polymorphisms (SNPs) that were associated with RBA within the evaluation set subjects. Multiple SNPs in a 2-Mb region on Chromosome 17 showed significant association with RBA (i.e., p-value < 5E-8). Figure 6 is the Manhattan plot showing association p-value between SNPs and relative brain age across the genome. Supplementary Table 3 lists the raw RBA-association p-values of 538,477 SNPs under analyses (doi: 10.5281/zenodo.3496206). A SNP located in MAPT gene showed the most significant association with RBA (SNP ID: Affx-13929237, p-value = 6E-9; see Supplementary Figs. 11, 12). We further built regression model to check if there was interaction between this SNP and smoking or alcohol consumption amount in association with RBA. The interaction term appeared to be insignificant in the model.

We also investigated the association between the dosage of APOE ɛ4 risk allele, a major Alzheimer’s disease risk factor, and RBA. We found that subjects with two copies of APOE ɛ4 risk alleles had slightly higher RBA than subjects with zero or one copy of risk allele (Supplementary Fig. 13). However, the association between APOE risk allele dosage and RBA was only nominally significant (p-value = 0.03) and didn’t reach significance threshold of genome-wide association studies.

## Discussion

Here we analyzed the brain imaging data collected for 17,308 UK Biobank subjects. We derived RBA metric using training set subjects, and further investigated the association of RBA with smoking, alcohol intake, and genetic variants using evaluation set subjects.

In our analyses, we first calculated PBA of a subject based on structural MRI data and then derived RBA, a metric that describes a subject’s PBA relative to peers. RBA was calculated as the difference between PBA and EPBA (i.e., RBA = PBA − EPBA; see the methods section for details) of a person. As a comparison, in other studies where PBA was derived based on regression model, the difference between PBA and CA (PBA − CA, or BrainAGE) was used to indicate the brain aging status^23,25,26^. We observed that due to regression dilution, older subjects tend to have negative values of PBA − CA, while younger subjects tend to have positive values of PBA - CA (Fig. 1 and Supplementary Fig. 3). As a comparison, RBA was independent of CA. At all age ranges, roughly half of the subjects had positive RBA and half of the subjects had negative RBA (Fig. 1 and Supplementary Fig. 4). Therefore, the association analyses of RBA with lifestyle and genetic factors are not affected by chronological age.

We showed that subjects with positive RBA performed worse in various cognitive functions while subjects with negative RBA performed better. A relevant study reported that the biological brain aging accelerated in patients with cognitive impairment than in normal subjects^27^. Our findings further demonstrated that even among cognitively normal subjects, there was association between advanced brain age and declined cognitive function. We noticed that while the correlation between Fluid intelligence score and RBA was statistically significant, it was not strong. That is due to three main reasons. First, although aging is associated with declined cognitive function including Fluid intelligence, there are a lot of noises in this association (please also refer to our response to reviewer’s 4th question). For example, researchers using UK Biobank have reported that there was a weak association between chronological age and Fluid intelligence score^32^. Second, RBA is independent of the chronological age. Therefore, although we expect that subjects with positive RBA to have worse cognitive function and subjects with negative RBA to have better cognitive function, we also acknowledge that subjects with the same RBA have a wide range of chronological age, causing large variation of Fluid intelligence score. Therefore, it is expected that RBA had a weak, yet significant, correlation with cognitive function. Third, subjects included in our analyses are cognitively normal. The association between RBA and cognitive function might be relatively weaker within these cognitively normal subjects as compared to a study in which subjects range from cognitively normal, mildly cognitive impaired, and severely cognitively impaired.

Our analyses of smoking and RBA indicated that subjects who had smoked on most or all days had a significantly higher RBA compared to subjects who smoked less often. That was consistent with previous studies, which showed significantly greater rate of atrophy in certain regions of the brains of smokers^5–7^. Our data also showed insignificant difference of RBA among the subjects who smoked occasionally, only tried once or twice, or abstained from smoking. This suggests that the detrimental effect of smoking on brain aging occurs mainly among those who smoked on most days.

Our analyses of alcohol intake frequency and RBA indicated that subjects who drank daily or almost daily had a significantly higher RBA compared to those who drank less frequently. Our finding was consistent with previous studies, which showed that heavy alcohol consumption was detrimental to the brain^8,9,33^. We did not find significant RBA difference among subjects who drank alcohol less frequently or those who abstain from drinking. It has been reported that a small dose of alcohol is associated with a reduced risk of cardiovascular disease, coronary heart disease and stroke^34–36^. Moreover, cardiovascular health and brain health are related. Researchers have found that cardiovascular risk factors like hypertension and heart disease are associated with increased brain white matter abnormalities and brain atrophy^12,37^. Therefore, a small amount of alcohol may have certain beneficial to brain health through contributing to the cardiovascular health. Gu *et al*., have reported that light-to-moderate total alcohol intake was associated with larger total brain volume in elderly subjects^38^. Nevertheless, our results didn’t show RBA difference among subjects who drank alcohol less frequently or those who abstain from drinking. We also acknowledge that our observation would need to be further validated using an independent data set.

We also found genome-wide significant association between SNPs within a 2 Mb locus on chromosome 17 and RBA. The SNP showing the most significant association is located in MAPT gene. Previous studies also showed that mutations in MAPT, which encodes tau protein, are associated with dementia and Parkinson’s disease^39^. Therefore, although the SNPs showing genome-wide significant association with RBA are located in a locus that covers multiple genes, it is likely that MAPT gene is a functional gene for brain aging. Further wet lab experiment focusing on MAPT gene and its regulatory region may be carried out to understand the etiology of brain aging. On the other hand, in previous studies, researchers have identified SNPs that showed genome-wide significant association with specific brain morphometrics. For example, SNP rs7294919 (candidate gene TESC) was associated with hippocampal volume; SNP rs945270 (candidate gene KTN1) was associated with putamen volume; SNP rs10784502 (candidate gene HMGA2) was associated with intracranial volume^40,41^. It is possible that since brain age was a summary statistic of the morphometrics of multiple brain regions, the associations between SNPs and specific brain regions did not get reflected. Although we only found SNPs on the 2 Mb chromosome 17 loci showing genome wide significant association with RBA, the SNP-level RBA association p-values can be used for future meta-analyses, where results from multiple genetic association studies are combined for identifying potentially more significant SNP-phenotype associations.

Several studies had been done previously to inspect the association between APOE ɛ4 risk allele, a major genetic risk factor for Alzheimer’s disease (AD)^42,43^, and brain age. Cole *et al*.^15^ looked at the association between APOE ɛ4 status and brain-predicted age difference (PAD) in 669 elderly subjects and reported no association between these two variables. Another study of 30 individuals with Down syndrome reported that APOE genotype did not significantly influence brain-PAD^16^. Lowe *et al*.^17^ reported that APOE ε4 status did not have significant association with Brain Age Gap Estimation (BrainAGE) in healthy subjects, patients with AD or mild cognitive impairment. However, they did observe association between BrainAGE changing rates and APOE ε4 carrier status. In our analyses, we found that subjects with two copies of APOE risk alleles had slightly higher RBA than subjects with no risk allele or only one copy of risk allele, although the effect was not statistically significant. Therefore, the effect of APOE risk allele on brain aging is probably not strong within cognitively normal subjects.

Our study has some limitations. First, we used a linear regression model with LASSO to produce PBA based on structural MRI data. More sophisticated statistical approaches such as using principal component analyses for dimension reduction before LASSO regression, or using neural networks may help to improve the accuracy of PBA. Also, the combination of structural MRI and other types of brain imaging data (e.g., functional MRI, diffusion-weighted MRI) may help to improve the accuracy of PBA. A more accurate PBA would allow better estimation of RBA. Second, in our study, we investigated the association of brain age with tobacco smoking and alcohol consumption. Besides smoking and alcohol consumption, various environmental factors may be associated with brain age. For example, physical exercise and meditation had been reported to be associated with lower brain aging level^44,45^. Further, genetics also affects brain aging^14^. Therefore, the variation of RBA that can be explained by smoking and alcohol drinking amount was small (as reflected by the small R-squared in the regression model for quantifying the association of RBA with smoking and alcohol drinking amount). More studies can be done to help fully understand the factors associated with brain age. Third, we chose to use pack-years and grams of alcohol intake per day for assessing the smoking and drinking amount. There are alternative measurements for assessing smoking and drinking amount, which may yield slightly different findings^46,47^. Fourth, it is possible that more genetic variants that have strong effect on RBA do exist. However, these genetic variants may be missing from the current genotyping platform and are not detected through current analyses. Fifth, genetic predispositions are known to affect smoking and alcohol drinking behavior. For example, SNPs located in the region of alcohol‐metabolizing enzyme genes are significantly associated with alcohol dependence^48^. A SNP located in the nicotinic receptor gene is significantly associated with number of cigarettes smoked per day^49^. Therefore, it is possible that genetic variants affect alcohol and nicotine consumption and indirectly affect the RBA. Sixth, a larger sample size would increase the power for identifying factors and interaction among them that are significantly associated with a specific trait. With increased number of UK Biobank subjects for whom brain-imaging data are available, a future study may reveal more about factors associated with brain aging.

In sum, we studied the association of brain age with smoking, alcohol consumption, and genetic variants using the data collected for 17,308 cognitively normal UK Biobank subjects. These results provided useful insights into how brain aging is associated with smoking and alcohol consumption. Our analyses only identified genetic variants within a 2 Mb locus on chromosome 17 to be significantly associated with brain aging. Further studies potentially with even larger sample sizes will be needed to provide a clearer picture of factors associated with brain aging.

## Materials and Methods

### Overview of UK biobank project

The UK Biobank recruited ~500,000 subjects in the United Kingdom^50^. The participants have provided blood, urine and saliva samples. All participants have been genotyped. 20,000 participants scanned as of August 2018 were included in our study (including brain, heart, abdomen, bones and carotid artery). All participants had provided informed consent. The present analyses were conducted under data application number 25641.

### Magnetic resonance imaging (MRI) data

Details of the structural brain MRI data, such as imaging hardware and acquisition protocols, are described elsewhere^51,52^. In our analyses, quality controlled structural MRI data was obtained for 21,345 subjects. We excluded 1,222 (5.7%) subjects with brain and nervous system related illness, including cognitive impairment, neurological disorders or stroke, etc. (see Supplementary Table 4 for the list of diseases based on which subjects were excluded from our analyses). We further excluded 2,815 (13.2%) subjects with non-European ancestry (according to both self-reported ethnicity and principal component analyses on the genetic data). Brain imaging data of 17,308 subjects were used in our analyses. The age range of these participants is between 45.2 years and 80.7 years.

Brain morphometrics, including volume of cortical, subcortical and white matter regions, thickness and surface area of cortical regions, ventricle size, intracranial volume, etc., were obtained with FreeSurfer 6.0^53^ based on the T1 MRI brain scans, with the Desikan-Killiany atlas. FreeSurfer is documented and freely available for download online (http://surfer.nmr.mgh.harvard.edu/). Supplementary Table 5 lists the 403 brain morphometric measurements used in our analyses.

### Cognitive function

We used the data of cognitive function in its original form, which was collected during the visit for MRI scan. All subjects performed specific tasks as instructed by a computer. To be specific, the Fluid intelligence score indicates the capacity to solve problems that require logic and reasoning ability. It was based on subjects’ performance in identifying the largest number, calculating family relationship, interpolating word, etc. For the prospective memory task, subjects were asked to memorize a command in the middle of the cognitive tests and perform it at the end of the test. In the reaction time test, subjects were asked to press a snap-button when two cards displayed on the computer screen matched. Mean time to correctly identify matches was recorded. In the pairs matching test, subjects were asked to memorize the position of matching pairs of cards. The number of correct pairs identified was recorded. More details of the tasks for assessing cognitive function can be found on the UK Biobank website (http://www.ukbiobank.ac.uk/).

### Education

We used the information of education qualification collected during the visit for MRI scan. The qualification variable has multiple categories based on a British system. We collapsed it into two categories indicating whether or not a subject held a college or university degree, as used in the paper by Cox *et al*.^54^. There was a significant association between education and RBA (p-value = 0.009, Supplementary Fig. 10). Therefore, we also adjusted for education when assessing the association of RBA with smoking, alcohol consumption, and genetic variants.

### Tobacco smoking history and alcohol intake

We used the information of smoking history and alcohol intake status that was collected during the visit for MRI scan. The smoking and alcohol intake frequency categories used in our analyses were as reported in the UK Biobank questionnaire. The smoking pack-years was defined as the number of cigarettes smoked per day/20 multiplied by the number of years of smoking. The alcohol intake amount was calculated as described in the paper by Piumatti *et al*.^36^. Alcohol consumption per day for a specific type of drink was calculated as the number of drinks consumed per day multiplied by the number of grams of alcohol contained in one drink. The total amount of alcohol consumption per day was the summation of the alcohol amount from all types of drinks. More details can be found on the UK Biobank website (http://www.ukbiobank.ac.uk/).

### Genotype data

Details of the genotyping and genotype calling procedures are described elsewhere^55^. Quality-controlled genotype data was obtained for 538,477 autosomal SNPs genotyped for 11,900 evaluation set subjects. Our quality control on SNPs ensured that all SNPs had missing rate less than 0.02 and passed Hardy-Weinberg exact test (i.e., Hardy-Weinberg equilibrium p-value >= 1E-6). Quality control on the samples ensured that all subjects had genotyping rate greater than 0.98 and had heterozygosity rate within $\pm$3 standard deviation, had matched reported gender and genetic gender, and were of European ancestry (according to both self-reported ethnicity and genetic ethnicity based on principal component analyses). Related individuals (i.e., kinship coefficient >0.1) were further removed.

### Obtaining predicted brain age (PBA) and relative brain age (PBA) based on structural MRI data

Predicted brain age (PBA) is a metric describing how old a person’s brain appears based on a brain scan at a single time-point. Relative brain age (RBA) is a metric indicating if a person’s brain has experienced accelerated or decelerated aging compared to peers. It captures the deviation of a person’s brain structural aging from the population’s normal pace.

We trained a model for obtaining PBA and RBA based on MRI data using training set subjects. To be specific, we randomly split the brain imaging data of 17,308 subjects into training and evaluation sets. Our rationale for picking 30% (5,193) of the subjects as the training set and the remaining 70% (12,115) as the evaluation set was to balance the need for accurately training a model to predict brain age and the need for a large number of subjects in the evaluation set for evaluating the association of RBA and the factors of interest.

The model for obtaining PBA and RBA is trained as follows. We first trained a model obtaining predicted brain age (PBA) based on MRI data using data of the training set subjects. To be specific, we built a linear regression model with Lasso regularization for predicting brain age using R package glmnet^56,57^. In the model, the chronological age was the response variable, and 403 brain quantitative measures derived using Freesurfer were used as predictors. During model training, the Lasso parameter, lambda, was selected based on an internal cross validation using glmnet. We did not do any pre-selection on the predictors, since the training set sample size was sufficiently large relative to the number of predictors in the model. The mean absolute error (MAE) between PBA and chronological age in the training set was 3.5 years. We observed that due to regression dilution^28^, the difference between PBA and CA (i.e., PBA - CA) was negatively associated with CA. The older subjects tended to have negative PBA − CA, while the younger subjects tended to have positive PBA − CA (See Fig. 2 and Supplementary Fig. 3). Therefore, after obtaining PBA for each subject, we further calculated RBA. RBA is defined as the difference between PBA and expected PBA given a subject’s chronological age (i.e., RBA = PBA- Expected(PBA|CA)). Here, Expected(PBA|CA)), or EPBA, was obtained through building a regression model where CA was the predictor and PBA was the response variable. In that way, RBA is independent of CA. At each age range, there were roughly half of the subjects with positive RBA and half of the subjects with negative RBA (Fig. 1, Supplementary Fig. 4). A subject with positive RBA has a brain that appears older than those of peers, while a subject with negative RBA has a brain that appears younger. Since we linear operations were used to derive RBA based on PBA and CA, the unit of RBA is year.

After training the model for obtaining PBA and RBA using the training set data, we applied it to the evaluation set and carried on association analyses.

### Quantifying the association of RBA with previous tobacco smoking amount and alcohol intake amount

We quantified the association between previous tobacco smoking amount, alcohol intake amount, and RBA using a two-step regression model adjusting for gender and education. We first built a linear regression model using data of 2,327 evaluation set subjects who previous smoked daily or almost daily and did not abstain from drinking alcohol. We then identified subjects with large Cook’s distance as potential influential observations (i.e., subjects with Cook’s distance greater than 3* the mean Cook’s distance of all the subjects). We excluded these influential observations, fitted a second linear regression model, and reported results based on the second regression model. In total, data of 2,174 non-influential observations were used in the second-step regression.

### Testing the association between genetic variants and RBA

We used PLINK^58^ linear regression model for genotypic test, adjusting for gender, education, and first five genetic principal components of ancestry, to test the association between SNPs and RBA.

## Supplementary information

Supplementary Figures

Supplementary Table 1

Supplementary Table 2

Supplementary Table 4

Supplementary Table 5
