# Supplementary material for: Association of relative brain age with tobacco smoking, alcohol consumption, and genetic variants
Source: Sci Rep. 2020 Jan 30;10:10. doi: 10.1038/s41598-019-56089-4 (PMC6992742; doi:10.1038/s41598-019-56089-4)
Supplement: Supplementary file 1 — Supplementary Figures [file 41598_2019_56089_MOESM1_ESM.pdf]

Supplementary Figures

Association of relative brain age with tobacco smoking, alcohol consumption, and genetic variants

Kaida Ning [a, b], Lu Zhao [a], Will Matloff [a,c] , Fengzhu Sun [b], Arthur W. Toga [a, *]

[a] USC Stevens Neuroimaging and Informatics Institute, Keck School of Medicine of University of Southern California, Los Angeles, California 90033, USA

[b] Molecular and Computational Biology Program, University of Southern California, Los Angeles, CA 90089, USA

[c] Neuroscience Graduate Program, University of Southern California, Los Angeles, CA 90089, USA

Corresponding author: Arthur W. Toga

* Corresponding author at: USC Stevens Neuroimaging and Informatics Institute, Keck School of Medicine of University of Southern California, 2025 Zonal Ave., Los Angeles, California 90033, USA. Tel.: +1 323 442 7246; Fax: +1 323 442 0137

E-mail address: Toga@loni.usc.edu

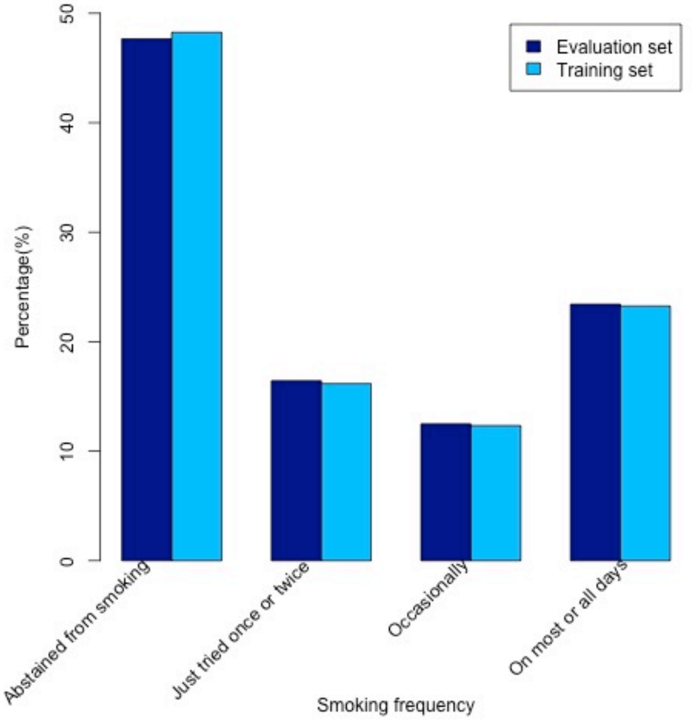

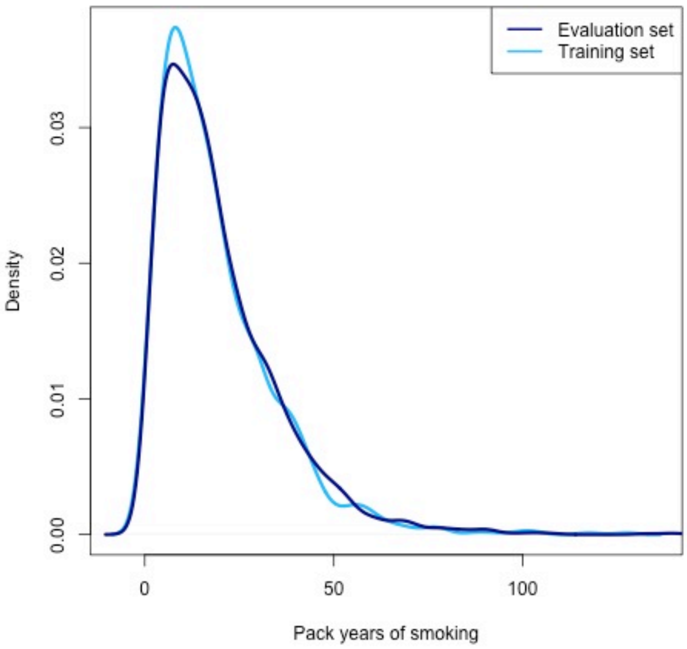

Supplementary Figure 1. Top: Tobacco smoking frequency in the evaluation and the training sets. Bottom: Tobacco smoking amount in the evaluation and the training sets.

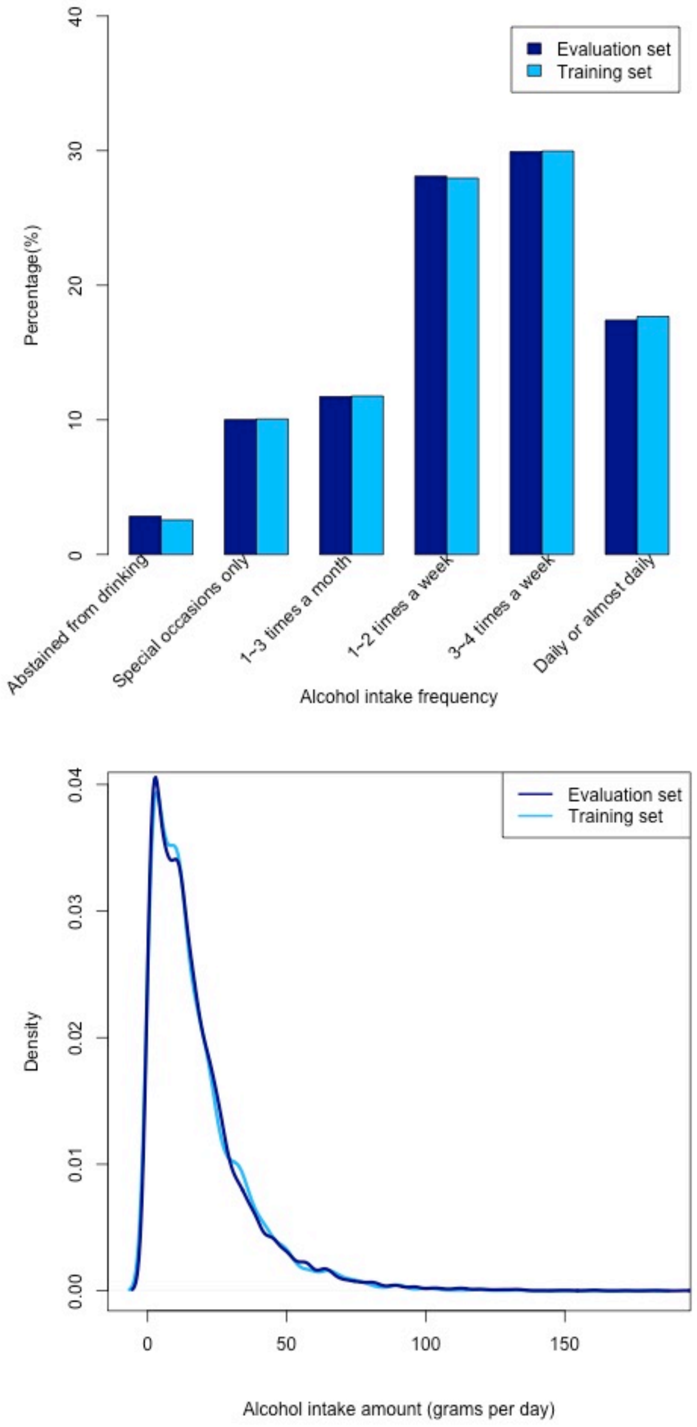

Supplementary Figure 2. Top: Alcohol intake frequency in the evaluation and the training sets. Bottom: Alcohol intake amount in the evaluation and the training sets.

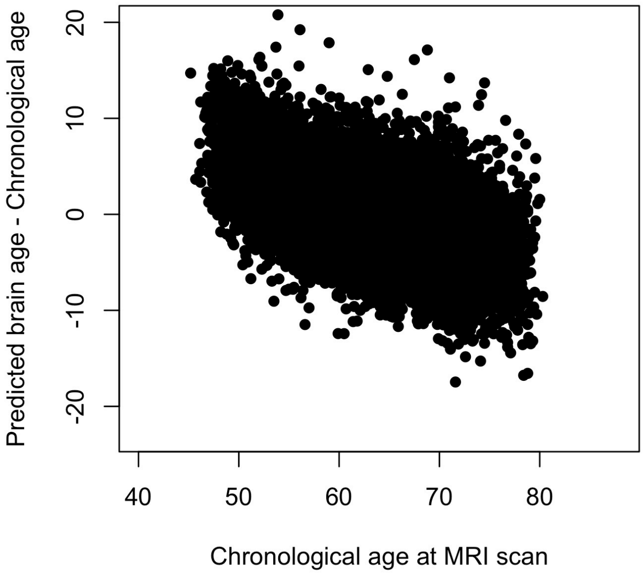

Supplementary Figure 3. Relationship between chronological age and the difference between predicted brain age and chronological age (PBA - CA, or BrainAGE) in the evaluation set.

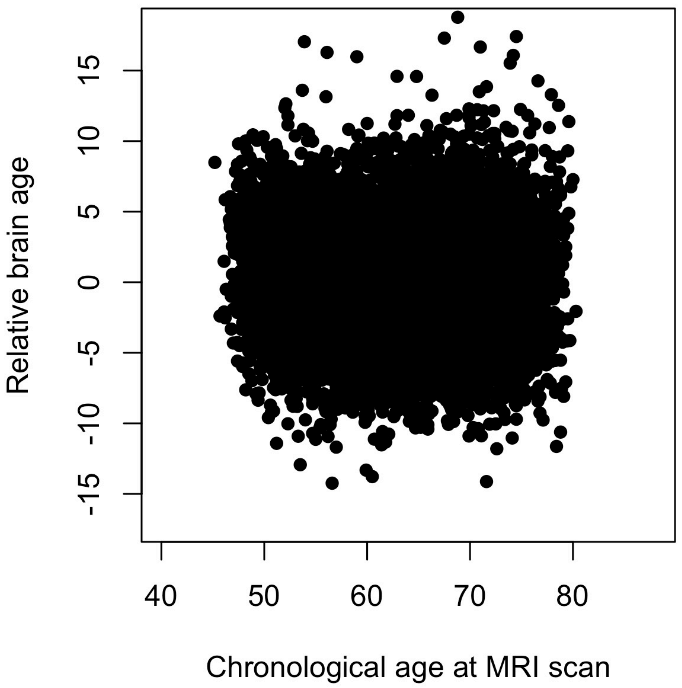

Supplementary Figure 4. Relationship between chronological age and relative brain age in the evaluation set.

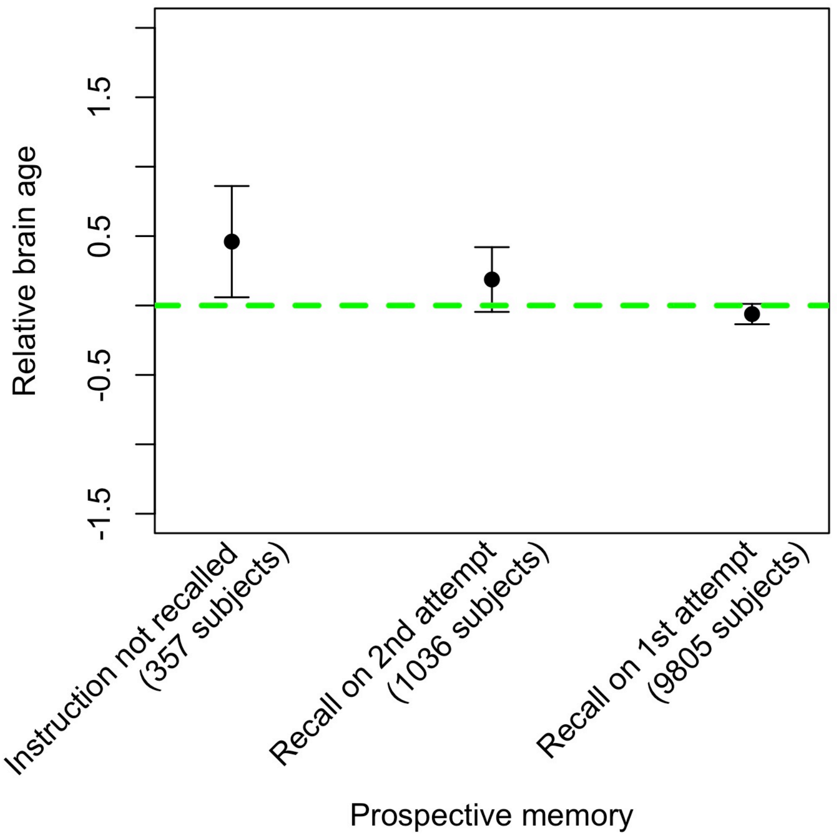

Supplementary Figure 5. Relationship between prospective memory and relative brain age (p-value = 0.005; R-squared=0.0007).

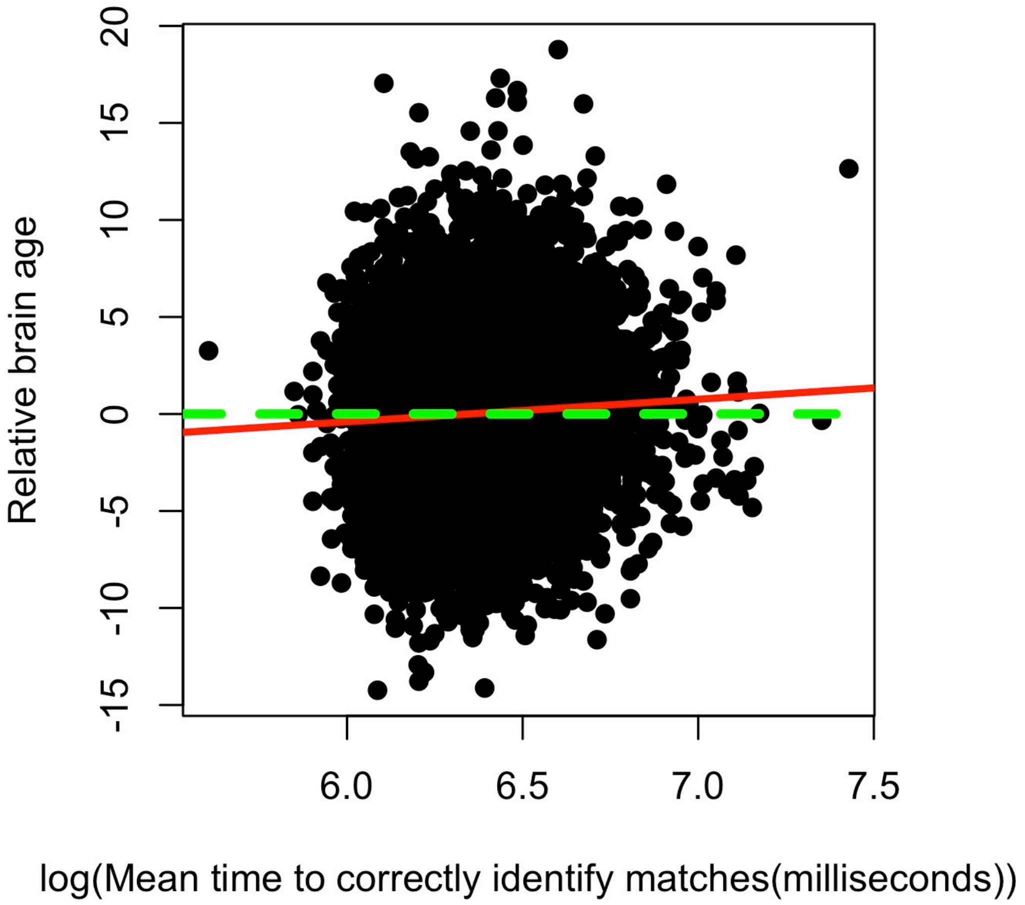

Supplementary Figure 6. Relationship between the time to correctly identify matches and relative brain age. Red line indicates the regression curve between the two variables (p-value = 2E-8; correlation = 0.04; R-squared = 0.003).

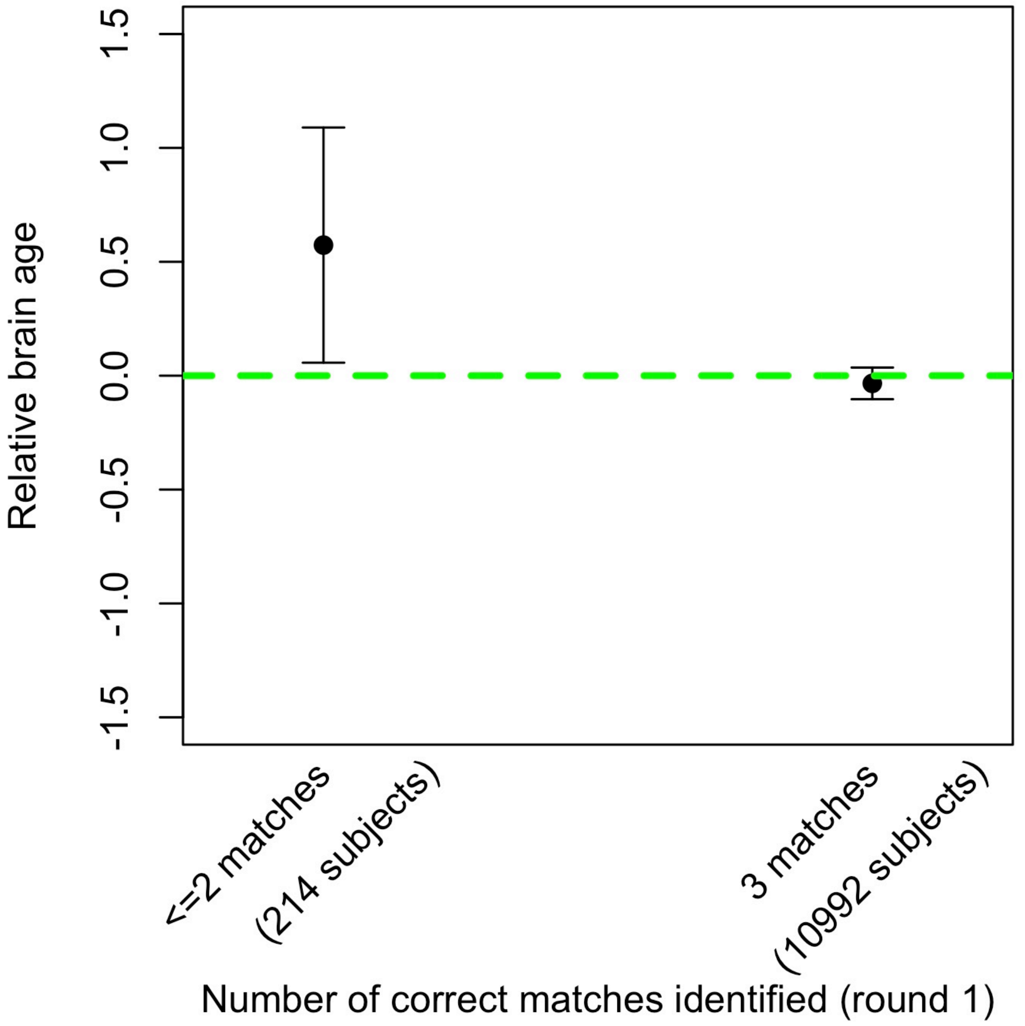

Supplementary Figure 7. Relationship between the number of matches correctly identified and relative brain age (round 1; p-value = 0.02; R-squared=0.0004). 203 subjects identified 0 matches, 10 subjects identified 1 match, 1 subject identified 2 matches. Those subjects were grouped together.

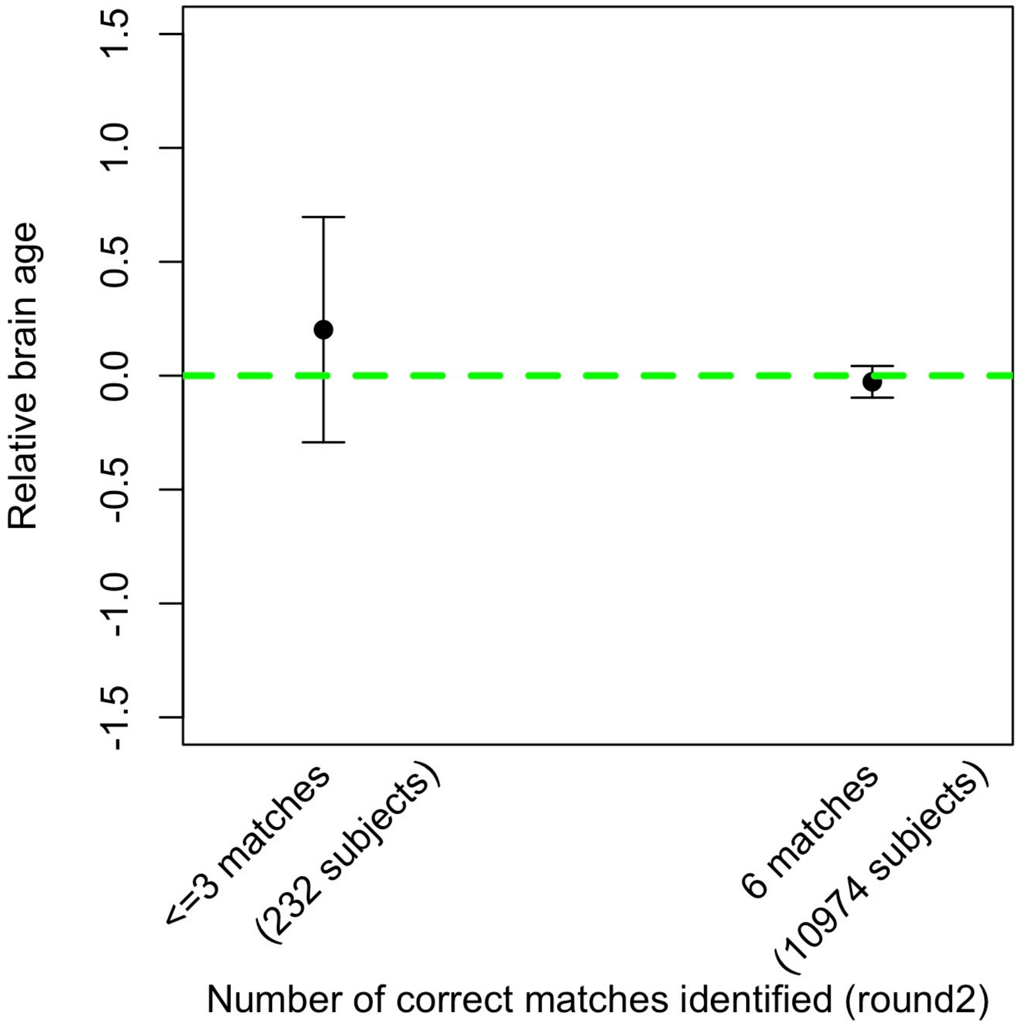

Supplementary Figure 8. Relationship between the number of matches correctly identified and relative brain age (round 2; p-value >0.05; R-squared=0.00001). 212 subjects identified 0 match, 15 subjects identified 1 match, 3 subject identified 2 matches, 2 subjects identified 3 matches. Those subjects were grouped together.

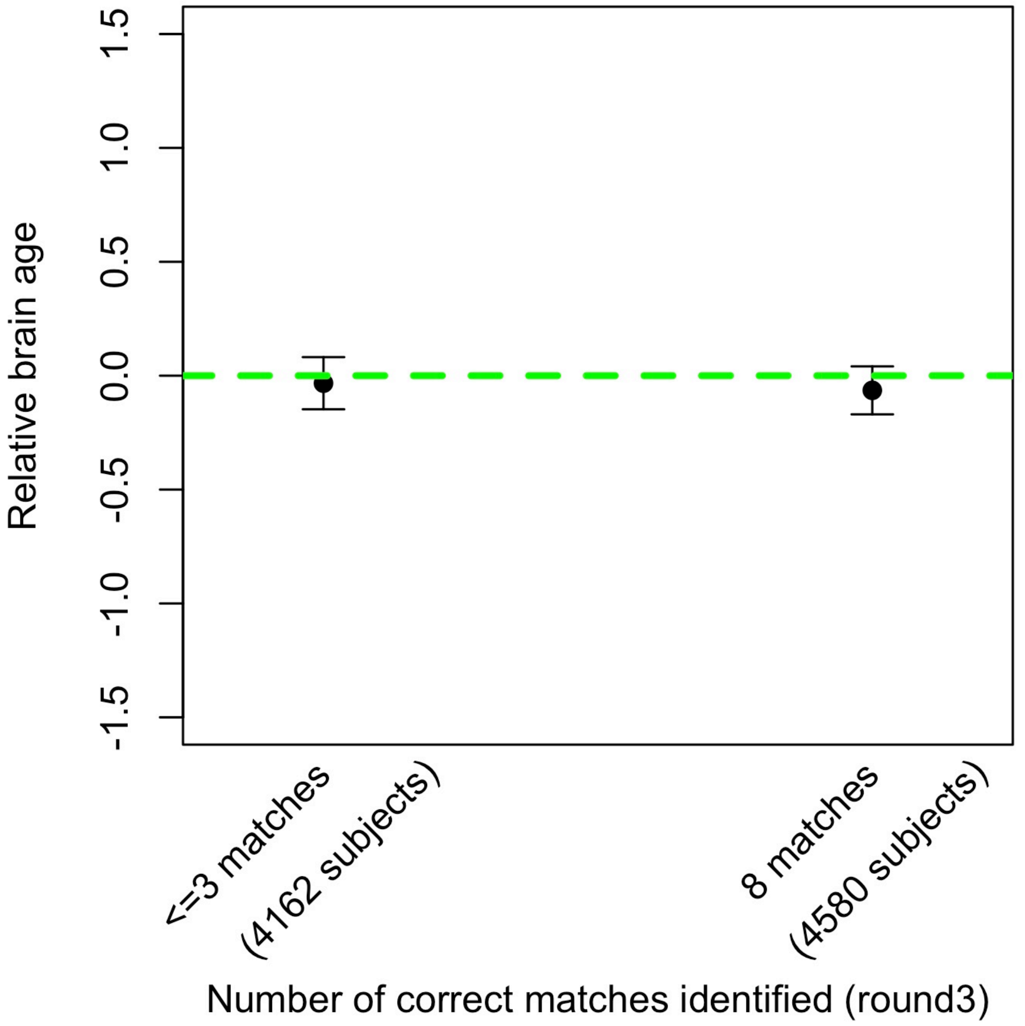

Supplementary Figure 9. Relationship between the number of matches correctly identified and relative brain age (round 3; p-value > 0.05; R-squared = 0.0001). 4,151 subjects identified 0 match, 7 subjects identified 1 match, 4 subjects identified 3 matches. Those subjects were grouped together.

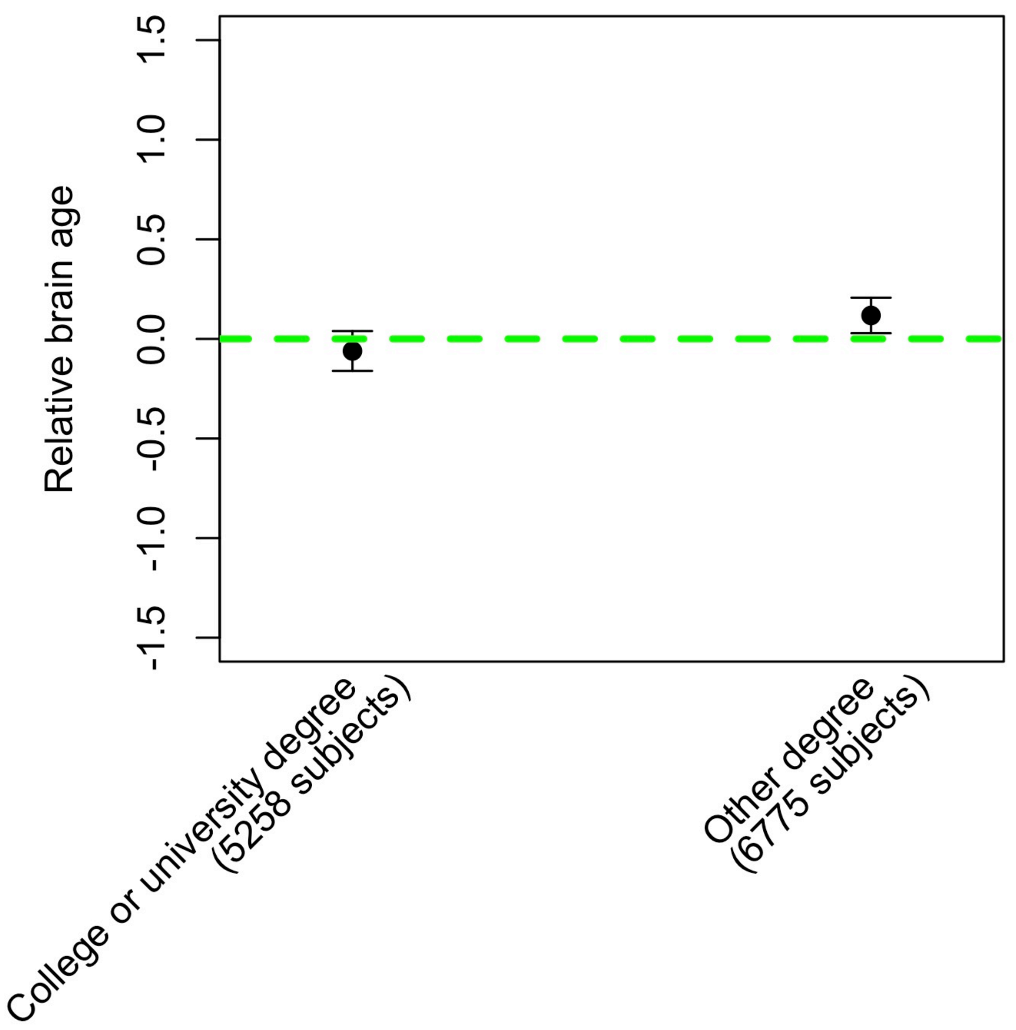

Supplementary Figure 10. Relationship between education and relative brain age (p-value =0.009; R-squared = 0.0006).

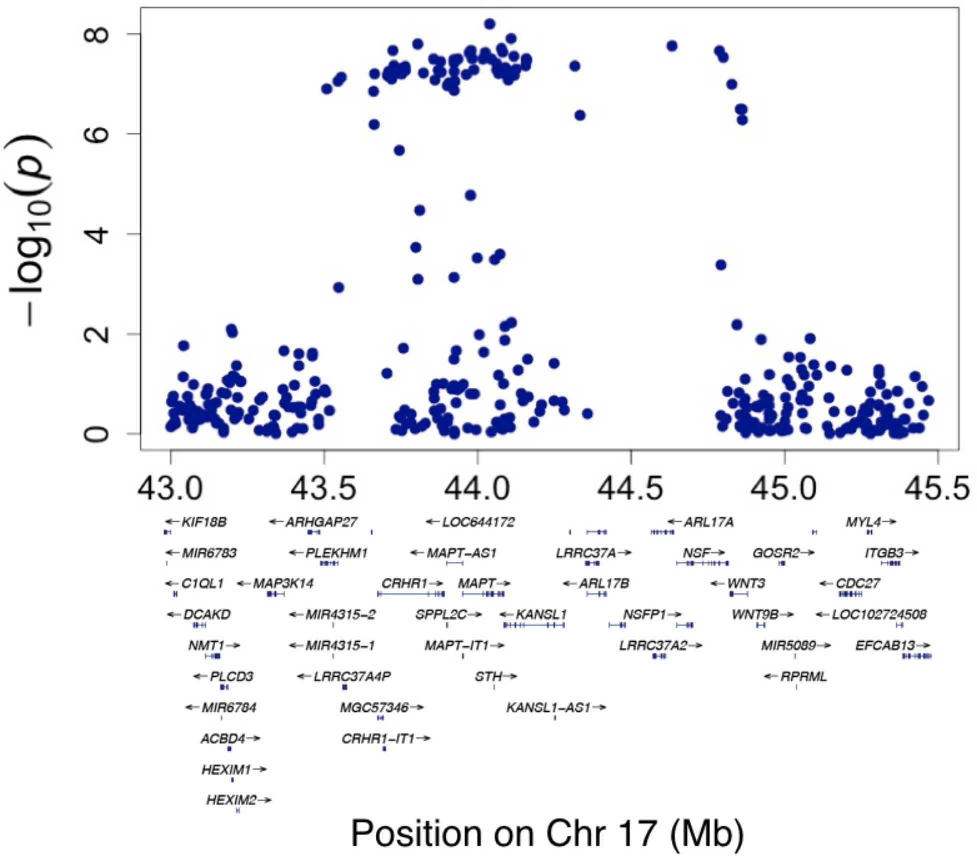

Supplementary Figure 11. Regional visualization of a 2Mb locus on Chromosome 17 where the SNPs showing most significant association with relative brain age are located.

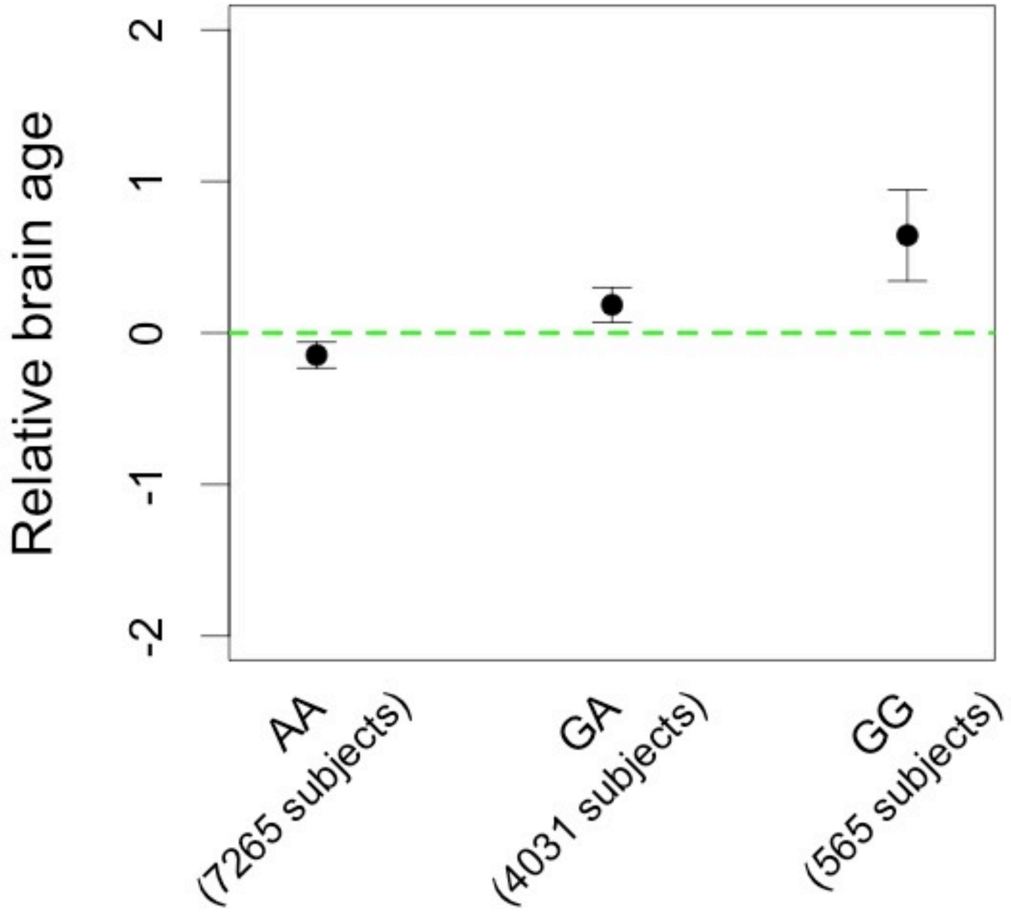

Supplementary Figure 12. Relationship between SNPAffx_13929237 and relative brain age (p-value = 6E-9).

.

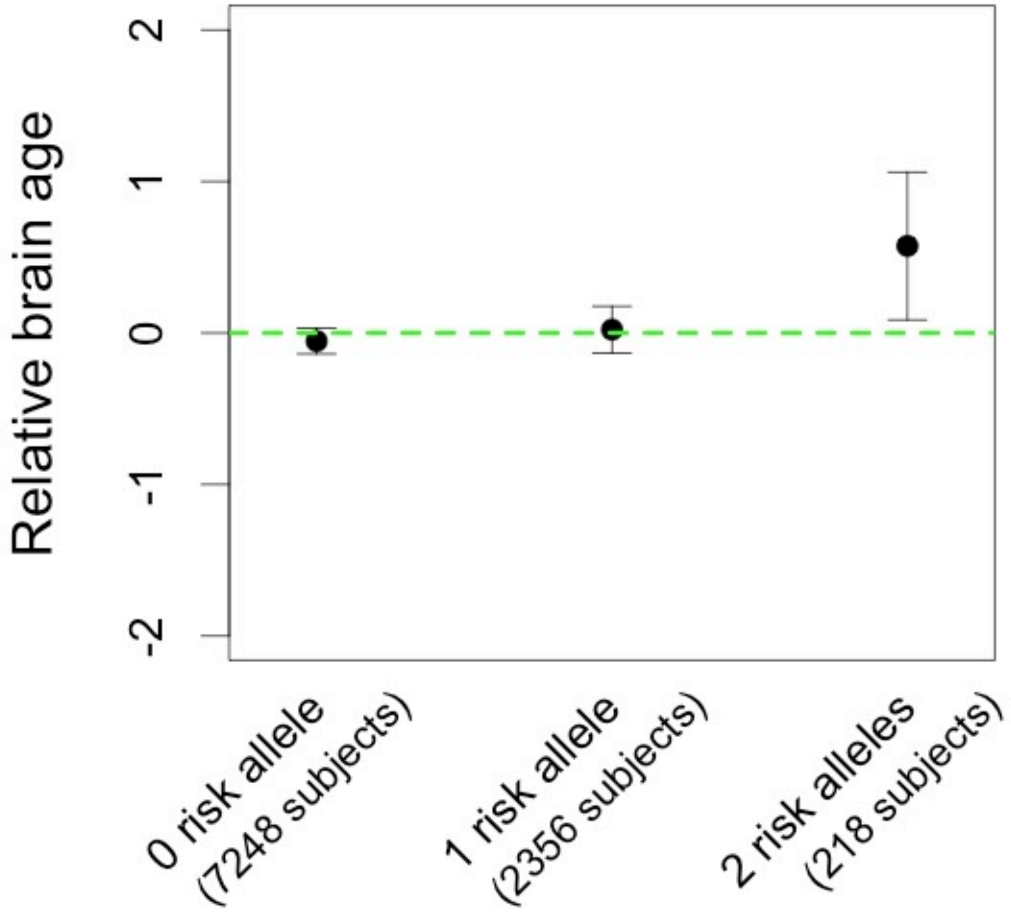

Supplementary Figure 13. Relationship between APOE ε4 risk allele dosage and relative brain age (p-value = 0.03).
